# Characterizing AIDS Drug Assistance Program Practices and Policies for Sustained Viral Suppression Using the Consolidated Framework for Implementation Research: Protocol for a Qualitative Study

**DOI:** 10.2196/90008

**Published:** 2026-04-16

**Authors:** Kathleen A McManus, Erin Q Rogers, Amber Steen, Amy Killelea, Tim Horn, Reanna Panagides, Steven Laymon, Jessica Keim-Malpass

**Affiliations:** 1 Division of Infectious Diseases & International Health School of Medicine University of Virginia Charlottesville, VA United States; 2 School of Nursing University of Virginia Charlottesville, VA United States; 3 National Alliance of State and Territorial AIDS Directors Washington, DC United States

**Keywords:** AIDS Drug Assistance Program, ADAP, viral suppression, Consolidated Framework for Implementation Research, CFIR, Ryan White HIV/AIDS Program, antiretroviral therapy

## Abstract

**Background:**

AIDS Drug Assistance Programs (ADAPs), a key part of the federally funded Ryan White HIV/AIDS Program, provide antiretroviral therapy (ART) to low-income and uninsured or underinsured people with HIV. As long as they meet federal regulations, jurisdictions maintain flexibility in implementing ADAPs, allowing for a range of operational and programmatic options, including eligibility criteria, formulary design for ART (and non-ART) drugs, and the nature and character of insurance support. These programmatic and policy decisions ultimately impact ADAP client outcomes, including engagement in care, differences in the populations benefiting from ADAP, and viral suppression rates. Despite ADAPs achieving an 85% national average viral suppression rate, this falls short of the 90% goal needed to end the HIV epidemic, and no formal evaluation has examined how specific operational or policy factors drive success across states.

**Objective:**

This qualitative study aims to examine how operational, organizational, and contextual factors shape the ability of ADAPs to improve viral suppression rates among people with HIV enrolled in ADAPs, specifically by identifying the mechanisms that facilitate high performance and viral suppression for all people with HIV. Results will inform how viral suppression outcomes could be improved through policy, program, operational, and organizational changes in ADAP design and implementation.

**Methods:**

Using a qualitative descriptive approach guided by the Consolidated Framework for Implementation Research, we conducted semistructured interviews with state-level ADAP leaders, operational and programmatic decision-makers, and staff. Interview questions covered 5 domains: leadership and staffing; operations, integration, and collaboration; formulary design and treatment; eligibility and enrollment; and innovation and implementation. Qualitative data from the interviews will be analyzed using deductive and inductive approaches along with situational mapping.

**Results:**

This study was funded in April 2023. In total, 33 individuals from 16 states have participated in this qualitative study, resulting in a participation rate of 31.4% (16/51). States from all 4 US census regions were represented. Interviews were conducted between February 12, 2025, and March 20, 2025. As of March 2026, data analysis is underway.

**Conclusions:**

We anticipate that the study will highlight features, organizational practices, and operational norms that contribute to the successes of ADAPs with high rates of viral suppression. Successful completion of this analysis will provide evidence to inform state and federal regulations, program development, resource allocation, and prioritization to advance the goal of helping people achieve viral suppression and interrupt HIV transmission, which is a vital public health objective. Using other data alongside our qualitative findings, we can provide context for the environment in which these programs or policies impact ADAP clients and the broader public health objectives of the Ryan White HIV/AIDS Program. Ultimately, we expect that disseminating these results will support improvements in viral suppression, enhancing both individual health outcomes and public health.

**International Registered Report Identifier (IRRID):**

DERR1-10.2196/90008

## Introduction

Despite the availability of safe and effective antiretroviral therapy (ART), the most recent estimate of viral suppression among people with HIV in the United States was 67% in 2023 [1]. To accomplish the goals of the federal Ending the HIV Epidemic initiative, all people with HIV, including those with low incomes, must have uninterrupted, affordable access to ART [2] to achieve sustained viral suppression, which is critical to individual health (reduced morbidity, comorbidities, and mortality) [3,4] and public health (preventing transmission of HIV and thereby reducing incidence) [5,6]. Additionally, viral suppression could reduce health care costs because each HIV infection averted saves up to US $1.2 million [7-9].

As a key part of the federally funded US Ryan White HIV/AIDS Program (RWHAP) health care delivery safety net, AIDS Drug Assistance Programs (ADAPs) provide free ART to people with HIV with low incomes, either through direct provision or ADAP-subsidized insurance plans [10,11]. People with HIV are eligible for ADAP support if they are uninsured or underinsured and have an income less than 200% to 500% of the federal poverty level, depending on their state’s eligibility criteria [11,12]. In 2023, ADAPs provided ART for 20% of people with HIV in the United States, with an annual national budget of US $2.5 billion [13]. Although people with HIV enrolled in ADAP achieved a higher average viral suppression rate (85%) [13] than the US average (67%) [1], this rate still falls short of the 90% goal needed to end the HIV epidemic [14]. Viral suppression rates also vary across states and among ADAPs, with a mix of external policy factors and internal programmatic decisions driving these differences. Nevertheless, the 85% viral suppression rate, as a national average across all ADAPs, demonstrates the value of intentionally focusing on delivering attentive care to low-income uninsured or underinsured people with HIV [13].

Since their inception, ADAPs have evolved to meet clients’ changing needs and have often expanded their formularies beyond the federal requirement of including at least 1 drug from each class of HIV antiretroviral medications [15]. This improves treatment effectiveness and adherence and addresses individual patient needs. As more ARTs have been approved by the US Food and Drug Administration, ADAPs have expanded their formularies to include new medications, such as single-tablet and long-acting injectable regimens [16]. Many have also incorporated non-ART medications to address comorbidities, such as hepatitis C and substance use disorders, and have developed premium and cost-sharing support to help clients maintain insurance and prescription drug coverage [17].

ADAPs have also adapted to a changing insurance landscape, where far more clients have public and private insurance coverage, both because of Affordable Care Act (ACA), Medicaid, and private insurance expansion and because of an aging population newly qualifying for Medicare [18]. ADAPs have expanded their programs to be able to not only provide free ART to low-income people with HIV without another coverage source but also assist insured people with HIV with their premiums and medication cost sharing [19]. Overall, ADAPs have demonstrated implementation agility by adapting to changes in their client base, the needs of people with HIV, innovations in treatment and care (including evolving formularies), and shifts in policy and funding [13]. With the expiration of pandemic-era continuous Medicaid coverage and significant changes to state Medicaid programs, state ADAPs will need to find new ways to preserve support for people with HIV and accomplish the broader aims of improving viral suppression rates for their clients [20].

Despite their demonstrated adaptability, differences in ADAP use [17] and outcomes [13] have emerged, and the drivers of these differences are poorly understood. Evidence indicates persistent differences in ADAP use, suggesting that smaller proportions of Black people with HIV and women are served by ADAP than White people with HIV and men, respectively [17]. ADAP performance for subgroups of people with HIV is shaped not only by client characteristics but also by the policy and operational contexts in which they function. A key challenge for ADAPs is how they collect, analyze, and act upon client data to assess program performance; identify differences in outcomes; and guide responsive interventions. The ways in which ADAPs approach this challenge will vary by jurisdiction, reflecting differences in context, policy, and infrastructure.

Although all state ADAPs operate under a common federal legislative and regulatory framework, implementation varies considerably [15]. Each ADAP has considerable latitude in how it structures its medication procurement and delivery systems, as well as how it formulates its day-to-day client service, case management, and communications practices [15]. These differences are present in several ways, including the extent to which ADAP assists insured people with HIV with premiums and cost sharing, program eligibility criteria (including income thresholds and recertification frequency), formulary composition (both ART and non-ART drugs), pharmacy network structure, and availability of mail-order drugs. States also vary in client mix and demographics, the percentage of clients supported through health insurance assistance vs full-pay ADAP, and the organizational placement of the ADAP within the broader state health department or social service agencies. Further variation exists in ADAPs’ relationships with the larger public health apparatus, including HIV surveillance and prevention efforts and state Medicaid offices, and in the degree of coordination with community-based and faith-based organizations that promote ADAP awareness, access, and enrollment. Programmatic decisions made by ADAPs also occur against a backdrop of budget constraints [21], with relatively flat federal appropriations for RWHAP over the past decade.

To date, no formal evaluation has examined how these factors and characteristics influence ADAP implementation or their capacity to improve viral suppression rates among people with HIV. This represents a key gap in the literature and a primary motivation for this study. The inner workings of ADAPs within states—how context, operational architecture, economics, and organizational practices translate into outcomes—remain to be described. The most recent qualitative study of ADAPs was performed in 2011, focusing on how ADAPs would adapt to the ACA [22]. Since the ACA’s implementation in 2014, both HIV care delivery and ADAP operations have evolved [13,23]. Additionally, the population served by ADAPs has changed, with new diagnoses increasingly occurring among people who are Black or African American and/or Hispanic or Latino. There are also increasing comorbidities among older people with HIV. Understanding how ADAPs adapt to these changing demographic and clinical needs is essential to supporting their mission of improving viral suppression and advancing toward the 90% goal of the Ending the HIV Epidemic initiative [2].

The objective of this research is to understand how operational, organizational, economic, and contextual factors shape ADAPs’ ability to improve viral suppression rates among low-income people with HIV. The theoretical framework guiding this qualitative research study is the Consolidated Framework for Implementation Research (CFIR) [24]. The CFIR is a flexible and useful framework for understanding policy implementation and the impact of innovation across a range of operational settings. Health care researchers have used the CFIR to understand organizational practices and policies, operational norms, and the challenges and contexts accompanying the implementation of innovation and the introduction of new systems. The value of the CFIR lies in its reach, enabling an understanding of how a variety of factors, including participants’ needs and characteristics, organizational culture and operational routines, relevant governmental or regulatory guidelines, and the character and quality of organizational leadership, shape or determine outcomes. The CFIR allows researchers to understand how a variety of context-specific variables impact how well a system or set of practices serves the clients, patients, and participants it is designed to serve. We seek to better understand how ADAP structures and processes integrate within heterogeneous state contexts.

**Figure 1:**
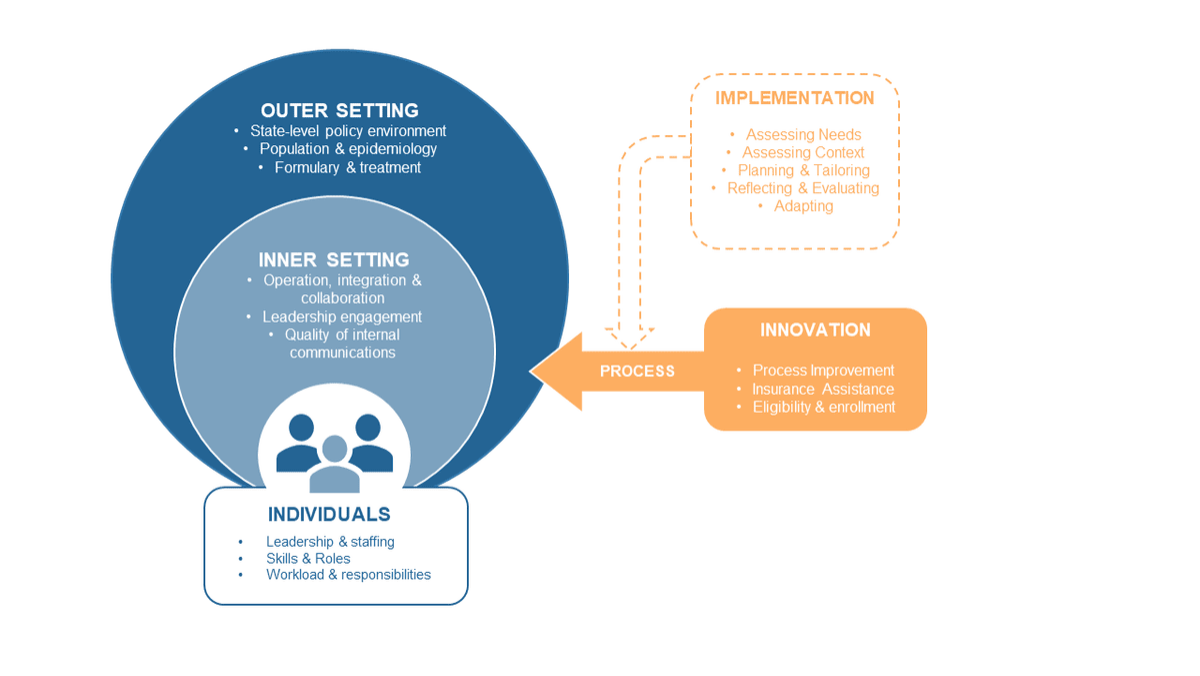
Application of the Consolidated Framework for Implementation Research to inform the design and analysis of qualitative interviews.

One feature of the CFIR as a conceptual instrument is the continued engagement of the instrument’s designers in ongoing efforts to refine and supplement the CFIR as a research and evaluative tool [24]. The codevelopers behind the CFIR have assembled data that provide a glimpse into how health care and public health researchers have used the CFIR to guide data analysis and to evaluate and design or redesign implementation strategies [24]. This results in a broad community of practice offering crowdsourced solutions to research, application, and evaluation questions. Figure 1 shows our CFIR model of the ADAP operational landscape.

## Methods

### Overview

This study takes a qualitative descriptive approach [25,26] to identify ADAPs’ programs and policies that could help ADAP clients achieve sustained viral suppression for individual and public health benefits. Enrollment was at the state level.

### Data Collection

Data collection involved individual semistructured interviews and was conducted via Health Insurance Portability and Accountability Act (HIPAA)–compliant videoconferencing to maintain the confidentiality of responses. Interviews were recorded and transcribed verbatim. Participants also responded to a brief survey focused on the characteristics of ADAP structures.

While the interview questions focused on ADAP structures and processes, some respondents may have offered insight into other aspects of the RWHAP and the general health infrastructure within the state, and we will incorporate these findings. Each interview consisted of open-ended questions organized into five parts covering five domains: (1) Opening questions focused on individuals and their roles and experiences in the operation and oversight of their state ADAP; this included questions about ADAP successes and challenges. (2) Operation, integration, and collaboration were discussed, which included understanding connections to other state agencies and health department personnel. We also assessed input from community stakeholders; connections to other social service providers; and interconnectedness with ADAPs in other states, the National Alliance for State and Territorial AIDS Directors (NASTAD), and other external partners, as well as how these are operationalized. (3) ADAP medication procurement and delivery policies were explored, including insurance assistance programs and processes shaping ADAP policy decisions, including questions about program eligibility, formulary design, and pharmacy network design. (4) Questions about eligibility criteria and enrollment policy, practices, and operational machinery included questions about state ADAP internal assessments of viral suppression rates and goals; differences in viral suppression among subpopulations who are disproportionately affected and/or historically underserved; and suggestions for ideal resources, policies, and programs that they think would improve viral suppression and reduce differences in outcomes for ADAP clients. (5) Concluding questions focused on innovation and implementation, which included program assessment, stakeholders involved in evaluating outcomes, data and data literacy challenges, and perceptions of the value of ADAP, as well as needed state or federal support, resources, or regulatory changes to implement best practices and improve health outcomes.

The interviews concluded with open-ended questions that invited participants to share additional thoughts. They will also be asked to identify any key stakeholders whom we should invite to participate in the study.

The study used maximum variance sampling to obtain qualitative interviews across a broad range of state ADAPs to maximize contextual insight. States will be selected based on the following key variables: (1) viral suppression rates [27] and (2) contextual variables, including region or geographic diversity [28], HIV prevalence [29], number of ADAP clients [12], state financial contributions to ADAP [12], and state Medicaid expansion status [30], as has been done by us [31] and others in previous work [22,32]. This sampling strategy will enable us to gain insight into a wide range of state ADAPs. These variables are critical as they represent external policy and resource factors (outer setting in CFIR) that constrain or enable ADAP operations.

From the 50 US states and the District of Columbia (n=51), state health department HIV leaders, including AIDS directors and ADAP directors and staff, were approached to participate in this study. The list of eligible participants (with their email addresses) is publicly available and was verified by NASTAD [33]. We used modified snowball sampling to recruit additional participants based on enrolled participants’ suggestions for key stakeholders in HIV care whom we should interview (eg, politically appointed health department personnel, community opinion leaders, and implementation team members), aligning with CFIR roles [27]. When more than 1 participant was interested in a state, we offered a joint interview or individual interviews.

The interview questions were drafted to uncover rich information about ADAP operations, policies, and practices, including eligibility criteria and re-enrollment requirements, organizational architecture and leadership, and participant and client profiles. For example, we anticipate learning about individual roles within ADAPs, staffing, and staff workloads; patterns of integration and cooperation with external partners and the wider community of actors conceptualizing and guiding ADAPs’ policies and practices; how state agencies and political and operational leadership cooperate and support ADAPs’ work; variations in program eligibility criteria and how criteria are adapted to respond to shifts in the level and character of need; and the ways in which innovations and new practices are implemented and adopted. Additionally, resources, including ADAP budget and revenue generation opportunities, are a key part of what drives operational and programmatic decisions, and these topics are covered as well. Table 1 shows the domains of qualitative interview questions and their alignment with the CFIR. The full interview guide is available in Multimedia Appendix 1.

**Table 1:** Domains of qualitative interview questions and alignment with the Consolidated Framework for Implementation Research (CFIR).

Domains	CFIR settings	Questions asked in the survey and key analytical concerns
Leadership and staffing	Individuals	How many people work full time in your ADAP^a^? How many work part time? Describe the workload and responsibilities. Is work outsourced or assigned to external contractors? Do you have an ADAP advisory committee? What is the advisory committee’s composition? Does it meet regularly?
Operation, integration, and collaboration	Inner setting	What is the relationship with the state health department? Is it part of the same agency responsible for Ryan White HIV/AIDS Program Part B? Is it part of the same agency responsible for HIV prevention and surveillance? What is the role of politically appointed health department personnel? What medical and nonmedical casework is completed and what is the relationship between the two? What input do you receive from community stakeholders? What connections does ADAP have with other social service agencies or providers? What connections exist between ADAP and programs in other states, the National Alliance of State and Territorial AIDS Directors, and other external partners?
Operations	Outer setting	What ideal resources, policies, and programs would improve viral suppression for ADAP clients?
Formulary design and treatment	Outer setting	What is the formulary design for antiretroviral therapy? What is the formulary design for non–antiretroviral therapy medications? How are these decisions made? What is the allowed duration of medication supply? What is the pharmacy network design? Do you offer or require mailing of medications? Does the full-pay formulary design differ from the insurance assistance formulary design? How does ADAP obtain client-level data from clinics and laboratories? Do you have a process for clients to self-report laboratory results? How are data harmonized across various laboratories and clinics?
Eligibility and enrollment	Outer setting and innovation	What is the income threshold and the mechanisms used for determining it? What is the process by which eligibility is determined? How are clients notified about or screened for re-enrollment? How do clients lose ADAP coverage? How can they regain eligibility? What is this process? How can individuals regain eligibility, and what does this process involve? What is the process for enrolling in full-pay ADAP or ADAP-assisted insurance coverage?
Innovation and implementation	Innovation	Does your ADAP perform internal assessments of disparities in viral suppression related to race or other factors? Does your ADAP perform internal assessments of engagement in the ADAP? What are the data challenges, data system challenges, and data literacy issues for your ADAP? Who is involved in policy and implementation decision-making? Are there any recent or planned changes related to eligibility? How are these changes made?

^a^ADAP: AIDS Drug Assistance Program.

### Outcomes and Data Analysis

Analyses will include descriptive statistics, conventional qualitative content analysis [34], and situational mapping [31], in which themes are inductively derived from data [34]. Data analysis will combine inductive and deductive methods of qualitative content analysis, with deductive components guided by CFIR [34]. Specifically, we will develop an initial coding dictionary using relevant CFIR domains (eg, inner setting, outer setting, innovation, and characteristics of individuals). Segments of interview data will be coded to these domains to systematically identify implementation determinants, such as leadership engagement, resource availability, interagency coordination, data infrastructure, and policy constraints. This approach ensures that analysis remains theoretically anchored and allows examination of how multilevel contextual factors influence ADAP operations.

The remaining analyses will be divided between research team members, who have in-depth experience with both forms of qualitative content analysis used in the study. Analyses will be discussed at full team meetings. These will be conducted using Dedoose (version 10.0.59; SocioCultural Research Consultants LLC). Situational mapping will be used to visualize and analyze the complex interplay between ADAP (the “what” or innovation), organizational context (inner setting), external environment (outer setting), and resultant client outcomes (viral suppression) across different states, as guided by CFIR.

Research team members will maintain reflective journal entries throughout the research process. Methods to ensure rigor and trustworthiness [35] of the data analysis will include aspects of demonstrating (1) credibility (direct observation of online communication, iterative questioning, and frequent debriefing), (2) transferability (contextual review), (3) dependability (maintaining an audit trail), and (4) confirmability (bracketing and investigator triangulation).

We will use the Consolidated Criteria for Reporting Qualitative Research reporting criteria when we publish the primary analysis [36].

### Ethical Considerations

This study has received ethics approval from the University of Virginia Institutional Review Board for the Social and Behavioral Sciences (Protocol #5707; modification approved on December 1, 2025). NASTAD and JSI Research and Training Institute Inc are collaborating partners supporting implementation, analysis, and dissemination activities. University of Virginia Institutional Review Board–Social and Behavioral Sciences serves as the institutional review board of record. JSI obtained a supplemental exempt determination (institutional review board #24-56E) for the participation of its staff. All participants were adults. Prior to participation, the study purpose, procedures, confidentiality protections, and potential risks and benefits were explained to each prospective participant. Verbal informed consent was obtained, and participants were informed that their participation is voluntary and that they could decline to answer questions or withdraw from the study at any point without consequences. No financial compensation was provided for participation. In the dissemination process, we will not identify individual people or states.

## Results

This study was funded in April 2023. A small adjustment to the study aims was made in 2025, as requested by the National Institutes of Health. The original and revised aims can be accessed in the National Institutes of Health RePORTER database [37,38]. As of March 2026, 16 states have participated, including 33 individual participants in our qualitative interviews, often with more than 1 interview per state. The participation rate was 31.4% (16/51). Characteristics of participating interviewees (N=33) and states (N=16) are included in Table 2. More than half of the participants were ADAP-related administrators (16/33, 48.5%), followed by HIV-related state health department administrators (15/33, 45.5%) and other state health department administrators (2/33, 6.1%). Participating states were geographically diverse, with the largest share in the West (6/16, 37.5%), equal representation from the South and Midwest (4/16, 25% each), and the fewest from the Northeast (2/16, 12.5%). Regarding financial eligibility requirements, half of the participating ADAPs set their federal poverty level threshold at ≥500% (8/16, 50%), while the remaining programs were evenly split between 400% and 500% (4/16, 25%) and 200% to 400% (4/16, 25%). Interviews were conducted between February 12, 2025, and March 20, 2025. As of March 2026, analyses are currently ongoing and expected results to be published in 2026.

**Table 2:** Sample characteristics of study participants (individual and states).

Characteristics			Participants, n (%)
**Role of individual participants (N=33)^a^**			
	HIV-related state health department administrator	15 (45.5)	
	ADAP^b^-related administrator	16 (48.5)	
	Other state health department administrator	2 (6.1)	
**Geographic region of participating state (N=16)**			
	West	6 (37.5)	
	South	4 (25)	
	Midwest	4 (25)	
	Northeast	2 (12.5)	
**ADAP financial eligibility threshold (FPL^c^** **), 2025, of participating State (N=16)^d^**			
	>500% of FPL	8 (50)	
	400%-500% of FPL	4 (25)	
	200%-400% of FPL	4 (25)	

^a^Summarizes the roles of participants who completed interviews. Across categories, roles spanned varying levels of responsibility, including program coordinators, managers, and directors.

^b^ADAP: AIDS Drug Assistance Program.

^c^FPL: federal poverty line.

^d^For all states included, ADAP’s financial eligibility requirements as of January 1, 2025, were concordant across both full-pay medication programs and ADAP-funded insurance programs.

## Discussion

### Context for Anticipated Findings

The overall viral suppression rate in the United States is 67% [1], which contrasts with the higher rate of 85% among people with HIV who receive services from ADAP [13]. This difference highlights an important opportunity to leverage ADAPs to improve HIV outcomes for low-income people with HIV [39]. Additionally, as ADAPs are helping people with HIV achieve higher-than-average outcomes, part of the reasoning behind focusing on ADAPs is that getting more people with HIV into ADAP could help improve viral suppression rates in the United States. It could be a key component of ending the HIV epidemic in the United States.

Our study focuses on this opportunity by studying ADAPs—a critical safety-net system that provides HIV medications for low-income people with HIV, either through direct provision of medication or assistance with premiums and medication cost sharing. Our approach is both theoretically and conceptually innovative. Specifically, we situate our analysis within the broader field of implementation science using the CFIR to guide our interview guide and subsequent analysis. This framework enables us to identify the key factors and contextual conditions that facilitate or hinder the adoption of innovations designed to improve viral suppression among people with HIV served by ADAPs.

A variety of mechanisms, organizational structures, operational practices, eligibility criteria, and related factors contribute to the success of particular ADAPs in elevating their clients’ viral suppression rates, providing continuity of care, and accomplishing broader public health aims that guide their operations. This study, given its reach across a broad range of state ADAPs representing the variety of policies and practices permitted under the RWHAP, can help identify policies, practices, approaches, and organizational structures that promote better outcomes for individual ADAP clients and public health objectives of wider health care and civic effort.

Our qualitative methodology, when used within a broader understanding of the health care needs and objectives related to people with HIV, is designed to assemble rich operational-level details about the mechanisms, policy structures, eligibility criteria, and decision-making processes that shape how effectively and responsively ADAPs serve an evolving client base in a changing policy and health care environment (Multimedia Appendix 2). The dynamic nature of the landscape in which viral suppression efforts unfold requires a better understanding of how decisions are implemented, innovations are adopted, and health care professionals and organizational and political leaders collaborate to implement best practices and ultimately improve health outcomes.

Much of HIV research overlooks the real-world barriers to achieving viral suppression among subpopulations who are disproportionately affected, historically underserved groups, and low-income populations. Despite the availability of safe and effective ART, more than one-third of people with HIV in the United States remain with detectable viral loads [1]. More work is needed to ensure that the tools available to safely and effectively treat HIV are reaching those who need them [40]. Our team’s recent quantitative work demonstrates that while state ADAPs serve less than a quarter of people with HIV, almost a third of the viral suppression in the United States can be attributed to people with HIV accessing medications through ADAPs [41]. ADAPs are essential for ending the HIV epidemic in the United States, and recent experts have declared ADAPs a model for chronic disease treatment support [42].

We anticipate that ADAPs with high rates of viral suppression will share information about programs and policies that could be disseminated to improve viral suppression and reduce differences in outcomes for subpopulations who are disproportionately affected and/or historically underserved. Because we will also have data on aspects of the states (such as Medicaid expansion status) as well as viral suppression rates, we can provide context for the environments in which these programs or policies affect ADAP clients. The Health Resources and Services Administration and state ADAPs could use these results to inform future regulatory and funding priorities. Following completion of the interviews, there were additional cost-containment measures imposed [43,44]. Therefore, these interviews will serve as a critical baseline assessment prior to the implementation and evaluation of the impacts of these cost-containment measures. In close collaboration with NASTAD, findings will be disseminated to traditional academic audiences and translated into actionable policy recommendations. Dissemination to stakeholders will include the development of brief, accessible policy reports and presentations at key national- and state-level RWHAP-ADAP convenings to facilitate shared best practices and immediate translation into operational changes.

Despite the study strengths, several challenges remain. Perspectives are drawn primarily from state health department HIV leaders, ADAP leaders and staff, and key stakeholders, which may underrepresent frontline staff and client experiences and may be influenced by institutional framing or social desirability. Although we use maximum variance sampling, participation is voluntary, and some states may differ systematically from those that did not participate. Viral suppression is shaped by many factors beyond ADAP design, including the broader HIV care infrastructure and health system navigation, Medicaid policies, and social and structural determinants. Findings also reflect a dynamic policy period (eg, the end of the COVID-19 public health emergency and the related Medicaid continuous coverage unwinding, as well as delayed funding and defunding of state public health systems), which may affect generalizability over time. Finally, we will mitigate concerns related to generalizability using team-based coding, audit trails, reflexivity, and triangulation.

### Dissemination Plan

We will disseminate the results of this study through traditional academic channels, including peer-reviewed manuscripts and presentations at national HIV conferences. In addition, study findings will inform the development of policy reports tailored to federal and state partners. In collaboration with NASTAD, we will share results directly with ADAP leadership and program staff to support translation of evidence into practice. We will align dissemination products with the Translational Science Benefit Model for optimal translation to impact policy and health outcomes [45].

### Conclusions

This qualitative study will address a critical gap by characterizing the operational, organizational, and contextual factors that enable ADAPs to achieve sustained viral suppression. By identifying key mechanisms that drive success across diverse state contexts, this work is well suited to inform policy and program development aimed at improving the overall health of people living with HIV. Findings will contribute essential evidence to guide ADAPs, policymakers, and public health leaders in strengthening the nation’s HIV care safety net.

## Appendix

Multimedia Appendix 1 Interview guide.

Multimedia Appendix 2 Peer review report from PPAH - Population and Public Health Approaches to HIV/AIDS Study Section, Healthcare Delivery and Methodologies Integrated Review Group (National Institutes of Health, USA).

## Abbreviations

ACA: Affordable Care Act
ADAP: AIDS Drug Assistance Program
ART: antiretroviral therapy
CFIR: Consolidated Framework for Implementation Research
HIPAA: Health Insurance Portability and Accountability Act
NASTAD: National Alliance for State and Territorial AIDS Directors
RWHAP: Ryan White HIV/AIDS Program

## Data Availability

The datasets generated or analyzed during this study are not publicly available due to the sensitive nature of the interview content and the terms of participant consent but deidentified quotations are available from the corresponding author on reasonable request.
