# Supplementary material for: Characterizing AIDS Drug Assistance Program Practices and Policies for Sustained Viral Suppression Using the Consolidated Framework for Implementation Research: Protocol for a Qualitative Study
Source: JMIR Res Protoc. 2026 Apr 16;15:e90008. doi: 10.2196/90008 (PMC13133592; doi:10.2196/90008)
Supplement: Multimedia Appendix 1 [file resprot_v15i1e90008_app1.docx]

**UVA-NASTAD ADAP Study Interview**

**Introduction:**

Thank you so much for your time and participation. The overarching purpose of our study is to identify ADAP policies and programs that help clients achieve sustained viral suppression. As you well know, because ADAPs are state-designed, there are differences in the structure and programs associated with HIV care. We would love to learn how you define successes and barriers regarding ADAP policy decisions.

**First,** can you tell me a bit about the **structure of your ADAP** and its **staffing model**? Roughly how many people work full-time for ADAP, and how many work part-time?

What type of workload do the staff take on and what are they responsible for?

If there is outsourced or contracted work, what is contracted and why it is contracted? (Examples include pharmacy benefits manager, insurance benefit manager, medical benefits manager).

Can you tell me a bit more about how the ADAP is organized within or adjacent to the state health department?

Is it in the same agency that manages RWHAP Part B? Is it the same state agency that oversees the state HIV prevention and surveillance?

What kind of oversight does the state health officer (e.g., the State Health Commissioner), or other politically appointed health department personnel have over ADAP?

Is this officer involved with ADAP decision-making?

Are there other state health officials involved with ADAP decision making?

How do you view how your ADAP interfaces with other state agencies (for instance, Medicaid)? What is the nature of these connections? What are the benefits? What are the challenges?

Now we are switching gears to **clients served** in your ADAP. With ADAP eligibility ranging from 200-550% of the federal poverty level across the nation, how did your ADAP decide its income threshold for eligibility? Are there recent or planned changes as it relates to this income threshold?

Are there any other recent or planned changes as it relates to ADAP eligibility policies?

What is the process by which a person living with HIV is determined to be eligible and enrolled in the program?

Once a client is enrolled in ADAP, what is the process for enrolling in either full-pay ADAP (where ADAP provides medication access) or ADAP-assisted insurance coverage? If they are eligible for insurance coverage, are they assisted in other ways to complete the enrollment process for that coverage?

Can you describe the process for determining which components they receive from ADAP coverage?

Are there any guiding policy, procedures, or mechanisms in place that you use to maximize enrollment in Medicaid, Medicare, or commercial insurance?

How does your insurance assistance program generate rebate or program income revenue? Are there ways it is set up to maximize revenue?

Now moving to the other end of the spectrum, how are clients screened for re-enrollment?

Are application and eligibility determination processes and re-certification integrated and/or aligned across RHWAP Parts?

Besides the re-enrollment process, are there other ways that clients lose ADAP coverage? Does this happen often in your perspective? Are there times when they lose ADAP coverage for a period of time and then regain eligibility? If so, how does this process usually work? Are there any guiding policy, procedures, or mechanisms that are used to sustain enrollment in insurance once an individual is enrolled?

For ADAP policy decisions around eligibility, how was health equity considered?

We want to know a bit more about the **formulary design** of both ART and non-ART medications.

- What is the formulary design for antiretroviral therapy (ART)? How is it decided what drugs are covered?
- What is the formulary design for non-ART medications? Again, how is it decided what drugs are covered?
- What is the allowed duration of medication supply (30 days, 90 days)?
- What is the pharmacy network design?
- Do you offer or require the mailing of medications?
- Does the full pay formulary differ from the insurance assistance formulary?
- Can you tell me a bit more about the structure and how you think each structural decision impacts client access?

As it relates to formulary and drug decisions, how were those decisions made?

Did ADAP use a health equity lens to guide formulary decisions?

What input do you receive from community stakeholders, including clinicians and people living with HIV?

Do you think the current process is working well for your state?

What recommendations would you have?

Would federal guidance on formulary decisions be helpful?

Now we are switching gears to **decisions related to enrollment and case management**.

*[If case management is referenced earlier, the interviewer should acknowledge this and indicate that these questions are meant to more fully understand ADAP client engagement with case management]*

Many states have different relationships with and requirements for ADAP clients to engage with case management. We want to learn more about your states’ relationship with case management in the following questions.

Can you describe how your ADAP engages with case management?

Is there a requirement for ADAP clients to have case management?

If yes, what does that model of care look like?

How do you perceive the balance with patient autonomy and required case management?

How is medical and non-medical case management structured? Are there different levels of case management depending on acuity?

Who provides case management (for instance is it the health department staff or community-based organizations)?

Is the case management for ADAP services funded by the ADAP or the state Part B program?

We know that ADAPs **connect with many other social services**. Can you tell me a bit about how ADAP supports client linkage to and applications for other social services (Medicaid, HOPWA, housing services, SNAP, etc.)?

How else does your ADAP coordinate with other social services (Medicaid, HOPWA and housing services SNAP, etc)?

We know that Medicaid continuous coverage was unwinding for some states, which may impact demand for ADAP services in many states. What challenges have you encountered? What has been the impact on the program?

Does ADAP have policies in place that address clients who may move in and out of Medicaid (i.e., allowing clients to stay enrolled, but inactive, in ADAP)?

Now switching gears to the ADAP **coordination with the clinical team**.

How does your ADAP obtain client-level data from clinics, labs?

Do you have a process for ADAP clients to self-report labs?

How is data harmonized across various clinics and labs who might report values differently? Is someone manually entering this data? What are overall challenges in maintaining this type of individual level data?

What are the **data challenges** that exist in coordinating?

Specifically, data challenges, data system issues, data literacy of team?

Are there data reporting challenges- to HRSA, to NASTAD, to state legislators?

Do you have an **ADAP advisory committee**?

If so, what is the composition of the advisory committee and how is their input integrated into ADAP policy and action?

Do they meet regularly? And what is discussed during meetings?

**Now we want to know about how ADAP clients’ outcomes including viral suppression interface with some of these decisions.**

Specifically, how does:

- Does your ADAP perform internal assessment of engagement in HIV care? If so, is it used to inform for policy decisions?
- Does your ADAP perform internal assessment of engagement in ADAP program? If so, is it used to inform policy decisions?
- Does your ADAP perform internal assessment of viral suppression? If so, is it used to inform policy decisions?
- Does your ADAP perform internal assessment of disparities in viral suppression related to race or other factors? If so, is it used to inform policy decisions?

What are your suggestions for ideal resources, policies, and programs that would improve viral suppression for ADAP clients?

What are your suggestions for ideal resources, policies, and programs that would reduce disparities for ADAP clients?

How do you obtain information about best practices and learn from other states’ models of care?

**What are your suggestions for State/federal support needed**

- needed state or federal support or resources that would be helpful
- changes to state or federal regulation/oversight that would be helpful

HRSA has been encouraging state ADAPs to use their ADAP infrastructure to support state PrEP Drug Assistance Programs. Is that something your state is doing? If so, can you please tell us about that? If not, has your state explored this? Why or why not? Do you believe you can include a PrEP Drug Assistance Program in your state or offer a status-neutral approach to service delivery?

Finally, we want to reflections on the overall **value of ADAP**. What are the aspects of value that are not captured by numbers/quantitative reporting?

Is there anything else that we did not cover that you find is valuable to this discussion.

Now we are wrapping up. Are there any **ADAP stakeholders** from your state who you recommend that we invite to participate in the study? If so, please tell me their name and email address.

We have just a few questions to better understand the **workforce**.

What is your position and its relationship to the state ADAP?

How many years have you been in this position?

Have you been in other related positions? And if so, for how long?
