# Supplementary material for: Characterizing AIDS Drug Assistance Program Practices and Policies for Sustained Viral Suppression Using the Consolidated Framework for Implementation Research: Protocol for a Qualitative Study
Source: JMIR Res Protoc. 2026 Apr 16;15:e90008. doi: 10.2196/90008 (PMC13133592; doi:10.2196/90008)
Supplement: Multimedia Appendix 2 [file resprot_v15i1e90008_app2.pdf]

SUMMARY STATEMENT

(Privileged Communication)

PROGRAM CONTACT:
Dr Robin Huebner
240-627-3216
rhuebner@niaid.nih.gov

Release Date: 07/26/2022
Revised Date:

Application Number: 1 R01 AI170093-01A1
Formerly: 1R01AI170093-01

Principal Investigator

MCMANUS, KATHLEEN ANN

Applicant Organization: UNIVERSITY OF VIRGINIA

Review Group: PPAH
Population and Public Health Approaches to HIV/AIDS Study Section
AIDS - EXP. REV.

Meeting Date: 07/14/2022
Council: OCT 2022
Requested Start: 09/01/2022
RFA/PA: PAR20-036
PCC: A27E

Project Title: Viral Suppression for People with HIV with Low Incomes: Study of Disparities, Health Equity, and Best Practices
SRG Action: Impact Score:33 Percentile:24
Next Steps: Visit https://grants.nih.gov/grants/next_steps.htm
Human Subjects: XM-Human subjects involved - Multiple exemptions designated
Animal Subjects: 10-No live vertebrate animals involved for competing appl.
Gender: 1A-Both genders, scientifically acceptable
Minority: 1A-Minorities and non-minorities, scientifically acceptable
Age: 3A-No children included, scientifically acceptable

Project Year	Direct Costs Requested	Estimated Total Cost
1	441,486	717,244
2	442,011	718,097
3	470,750	764,787
4	464,283	754,280
5	465,368	756,043
TOTAL	2,283,898	3,710,451

ADMINISTRATIVE BUDGET NOTE: The budget shown is the requested budget and has not been adjusted to reflect any recommendations made by reviewers. If an award is planned, the costs will be calculated by Institute grants management staff based on the recommendations outlined below in the COMMITTEE BUDGET RECOMMENDATIONS section.
EARLY STAGE INVESTIGATOR
NEW INVESTIGATOR

1R01AI170093-01A1 MCMANUS, KATHLEEN

EARLY STAGE INVESTIGATOR
NEW INVESTIGATOR

RESUME AND SUMMARY OF DISCUSSION: This applicant seeks to determine what factors cause the racial/ethnic disparities observed in the viral suppression (VS) of persons with HIV (PWH) who use AIDS Drug Assistance Programs (ADAPs). This is a very significant undertaking as the results of this study could have a tangible public health impact by stemming HIV related morbidity and mortality among low income, mostly ethnic/racial minority PWH. The scarcity of research in this area and the number of PWH involved highlight the importance of this study. The premise of the study provides good support for the analysis planned, given evidence on impact of cost coverage for ART. The applicant and her collaborators form an excellent team, with complementary expertise and a strong record of collaboration. They have also engaged health departments that will place at their disposal rich, databases encompassing a diverse population across 6 states; they will also add a qualitative component to help identify the ADAP programs and policies that have a positive impact on viral suppression. This resubmission was responsive to prior critiques and the applicants have clarified issues raised in a satisfactory manner. The applicants have added an aim on cost-effectiveness, and more detail and justification. Reviewers commend the rigor of the methods proposed as well as the strength of the environment; they are confident this study can strongly contribute to ending the HIV epidemic. However, some weaknesses were noted which dampened enthusiasm for the application; among them were the following: There is a lack of detail regarding how the qualitative findings will be integrated with what is learned in other aims; the applicants allude to using data from the Ryan White program to extrapolate social determinants like homelessness; but social determinants were barely described in the application; the time frame for data collection is inconsistent, and the rationale for carrying out data acquisition to 2024 was not well-justified; analysis could potentially start sooner. Despite these weaknesses, a small minority of reviewers assessed the potential impact of the application as moderately strong and the greater majority as having potentially high impact.

DESCRIPTION (provided by applicant): People with HIV (PWH) need consistent access to care and antiretroviral therapy (ART) to achieve the sustained viral suppression (VS) critical for individual health (reduced morbidity, comorbidities, and mortality) and public health (preventing transmission of HIV). The United States (US) has significant geographic disparities in HIV VS for PWH with low incomes who rely on state AIDS Drug Assistance Programs (ADAPs), ranging 53% to 99%. ADAPs support 26% of PWH in the US with a $2.4 billion annual budget. As a key part of the US HIV healthcare delivery safety net, ADAPs provide free ART by direct provision or through ADAP-subsidized insurance plans. ADAP implementation varies widely because individual state ADAPs have decision-making flexibilities for health- care delivery programs and policies. Additionally, how ADAPs perform for different subgroups remains unknown. Understanding how ADAPs can optimize VS could reduce healthcare costs, because each HIV infection averted saves $402,000. To support ending the US HIV epidemic, our long-term goal is to use epidemiologic, including causal inference framework, and qualitative methods, to identify how to improve VS for all PWH, including those with low incomes, through healthcare delivery programs. With a focus on disparities and health equity, our over- arching research question is what specific healthcare delivery programs and policies for PWH with low incomes increase sustained VS rates and reduce VS disparities related to race/ethnicity? Our team has studied ADAPs since 2014. Our study results have been used to advocate to successfully change healthcare laws. The Principal

Investigator, the only researcher with federal funding to study this aspect of HIV healthcare delivery, and the interdisciplinary team will use expertise in novel causal epidemiology and qualitative methods. We have partner- ships with the National Alliance of State & Territorial AIDS Directors (NASTAD) and six state health departments providing access to multistate individual-level data (comprising 20% of ADAP clients; including ADAPs at VS rate extremes), and for a national study, we will interview AIDS/ADAP leadership. We will complete the following: Aim 1: we will decompose disparities in sustained VS related to race/ethnicity. We will quantify the mediating effect of a modifiable factor, ADAP healthcare delivery programs. Aim 2: we will quantify potential improvements in sustained VS for individual state ADAPs informed by state ADAP client mix. Using simulation for all state ADAPs, we will estimate the impact on sustained VS of ADAP clients changing to specific ADAP programs. Aim 3: we will perform interviews of AIDS/ADAP Directors across the nation. Using qualitative analyses, we will iden- tify the ADAP programs and policies that improve VS and improve health equity. Successful completion of this work will provide evidence to inform state/federal regulation and resource prioritization toward the goal of helping people achieve VS and interrupting HIV transmission. Our findings would allow state ADAPs, advocates, policy- makers, and Ending the HIV Epidemic initiative leaders to adopt best practices identified from our study and to develop new interventions (laws, guidance, funding) to optimize VS- translating data into policy and action.

PUBLIC HEALTH RELEVANCE: This is relevant to public health because sustained HIV viral suppression is a key health outcome, benefiting the individual's health (reduced morbidity, comorbidities, and mortality) and the community because sustained HIV viral suppression is associated with preventing HIV transmission. As the United States makes advancements in HIV care and treatment and gets closer to ending the HIV epidemic, we need to ensure that no one is left behind. People with HIV with low incomes who rely on state AIDS Drug Assistance Programs (ADAPs) experience geographic disparities in viral suppression, and for this population, we will identify the specific policies and healthcare delivery programs that increase sustained viral suppression rates and reduce sustained viral suppression disparities related to race/ethnicity.

CRITIQUE 1

Significance: 2
Investigator(s): 1
Innovation: 3
Approach: 3
Environment: 1

Overall Impact: The is a resubmission R01 from an early-stage investigator proposing to identify specific healthcare delivery programs and policies that improve sustained viral suppression and decrease racial and ethnic disparities in viral suppression for ADAP recipients. The resubmission is responsive to prior critiques, including removal of a cost-effectiveness analysis which was noted to be inadequately supported and of uncertain value, replacing the last aim with a nationwide qualitative analysis, the provision of greater detail in addressing missing data, quantitative bias analysis, inclusion of sustained viral suppression as an outcome measure, and justification of the causal framework and NNT thresholds as relevant for policy change at the state level. The significance of the work could be high given the breadth of state ADAP program coverage nationwide and the rigor of the prior research performed by this team. The ESI PI and co-Is are well positioned to complete the work given their robust experience and relationship with ADAP data and implementers, as well as their complementary expertise. The research environment is excellent. Strengths in the approach include use of multiple, heterogeneous state data, both client and state aggregate level analyses to interrogate the impacts of healthcare delivery programs on racial disparities in viral suppression as well as overall viral

suppression by state using client case mix, and rigorous analytical methods. There is a lack of framework or process of the quantitative and qualitative analyses will be strategically integrated / interpreted and the performance of the qualitative Aim 3 prior to the first 2 aims may provide key contextual information on healthcare delivery program implementation that could inform the quantitative analysis. These contextual / implementation issues may be key to understanding why ADAP subsidized health insurance, despite being less costly/client and associated with better VL suppression is not utilized broadly. Overall impact is high.

1. Significance:

Strengths

- Given the breadth of State ADAP programs coverage (26% of PLWH at an annual budget of $2.1 BN) the proposal to improve viral suppression and reduce racial and ethnic disparities through optimizing successful state policies can have a significant impact on achieving EHE goals.
- Prior work performed by the investigators analyzing ADAP data has found that states providing ART to patients through ADAP subsidized insurance plans had higher rates of VL compared to direct ADAP provided ART, underscoring the importance of healthcare delivery programs on clinical outcomes and supporting the proposed analyses.
- If successful, the study may inform state policy makers on best practices for ADAP programs and policies.

Weaknesses

- None.

2. Investigator(s):

Strengths

- Dr. McManus is an accomplished ESI, having received a F32 and K08, the latter being the sole Federal award dedicated to the impact of ADAPs on viral suppression. She has partnered with multiple state ADAP programs and is well positioned to lead the proposal.
- The co-investigator team has complementary expertise (epidemiology, causal analysis, qualitative methods, and ADAP policy), and a history of collaboration / mentorship, including some co-authorship on multiple manuscripts.
- Team includes NASTAD senior directors.
- The addition of two senior level scientists to provide “managerial advice for large complex research programs” is a response to a prior critique.

Weaknesses

- None.

3. Innovation:

Strengths

- Analysis of multistate ADAP data and state policy impacts is moderately innovative given the scarcity of existing research and the use of causal analysis.

Weaknesses

- None

4. Approach:

Strengths

- A history of robust engagement of state stakeholders, including state health departments, as well as the inclusion of NASTAD directors as co-investigators is a strength, with benefits currently manifested in existing and in process DUAs with ADAP programs.
- The states selected for quantitative analyses (AZ, FL, HA, SC, TN, and VA) are heterogeneous in terms of client demographics, program characteristics, average VL suppression and state participation in Medicaid expansion, providing the opportunity to evaluate the impact of policies.
- Choice of causal inference framework, outcome measures, methods to deal with missing data and quantitative bias analysis are justified and processes are well described.
- Use of multi-state individual level and state-level aggregate analyses are complementary in the aims of understanding the impact of delivery program impact on racial disparities in viral suppression and overall viral suppression based on other client characteristics / case mix.
- The qualitative work proposed in lieu of the CEA included in the initial submission is well described and will expand knowledge on the more granular aspects of individual healthcare delivery program implementation and best practices.
- Sex as a biological variable is considered through subgroup analyses by sex.

Weaknesses

- There is a lack of an approach or process of how findings from Aims 1+2 and Aim 3 will be strategically integrated / interpreted. Performance of the qualitative Aim 3 prior to the first 2 aims may provide key contextual information on healthcare delivery program implementation that could inform the quantitative analysis. Given ADAP-subsidized health insurance costing less per client and associated with higher viral load suppression, versus ADAP provision of ART only costing states more per client and associated with lower viral load suppression, lack of broader or universal uptake of the former must be associated certain either structural implementation barriers of the former, other client benefits of the latter, or some combination of the two.

5. Environment:

Strengths

- The facilities at UVA and Emory are excellent and provide the necessary support for the analyses proposed, including high performance computing, administrative support and a rich research environment.
- All six health departments are included and engaged, with DUAs in place or in process.

Weaknesses

- None

Study Timeline:

Strengths

- Not applicable

Weaknesses

Protections for Human Subjects

Acceptable Risks and/or Adequate Protections

- Aim 1 analyses have been determined as exempt by IRB. Aim 2 analyses of publicly available aggregate data does not require review. Aim 3 analyses have been determined as exempt by IRB.

Data and Safety Monitoring Plan (Applicable for Clinical Trials Only):

Not Applicable (No Clinical Trials)

Inclusion Plans:

- Sex/Gender: Distribution justified scientifically.
- Race/Ethnicity: Distribution justified scientifically.
- For NIH-Defined Phase III trials, Plans for valid design and analysis: Not Applicable
- Inclusion/Exclusion Based on Age: Distribution justified scientifically.
- Children < 18 excluded given differences in needs for healthcare delivery programs in the population. All races and genders will be included, based on ADAP population.

Vertebrate Animals:

Not Applicable (No Vertebrate Animals)

Biohazards:

Not Applicable (No Biohazards)

Resubmission:

- Very responsive, see above in overall impact.

Applications from Foreign Organizations:

Not Applicable (No Foreign Organizations)

Select Agents:

Not Applicable (No Select Agents)

Resource Sharing Plans:

Acceptable

Authentication of Key Biological and/or Chemical Resources:

Acceptable

Budget and Period of Support:

Budget Modifications Recommended (in amount/time)

CRITIQUE 2

Significance: 3
Investigator(s): 1
Innovation: 2
Approach: 3
Environment: 1

Overall Impact: The study proposes to identify ADAP healthcare delivery programs that help improve viral suppression for PWH. It focuses on reducing disparities observed by income and race/ethnicity. Aim 1 is a quantitative study to identify associations and determine causal mediators (ADAP healthcare delivery programs) of viral suppression disparities related to race/ethnicity by using client-level data from 6 state health departments. Aim 2 will quantify potential improvements in sustained viral suppression for individual state ADAPs from adoption of efficacious healthcare delivery programs identified in Aim 1. Aim 3 is qualitative analyses, through interviews with state AIDS Directors and ADAP Directors (61 interviews from at least 31 states), to identify ADAP programs and policies that are associated with viral suppression and health equity. This will help inform best practices for healthcare delivery to maintain higher levels of viral suppression. The premise is prior quantitative analyses conducted in 3 state health departments that show certain healthcare delivery programs achieving higher levels of viral suppression than others. The proposal has several strengths. Viral suppression among persons on ADAP programs vary significantly across states, identifying causal factors and mediators can inform healthcare delivery programs that could achieve 90% VLS, a key goal in Ending the HIV Epidemic. Excellent team of investigators and partnerships with key entities (NASTAD), led by an ESI with strong publication record on ADAP related studies. Though there are some concerns on the approach, stemming from the lack of data on SDOH, the overall approach attempts to make best use of what is available through quantitative and qualitative studies. If successful, it can have a high impact by informing relevant policy changes in ADAP programs across the country.

1. Significance:

Strengths

- Previous work showed ART through ADAP-subsidized insurance plans had more viral suppression than ADAP provision of ART only. Previous work used 2 years of data for 3% of PWH estimate non-causal associations. This new work will analyze 9 years of data for 20% of PWH with low incomes who rely on ADAPs. Use of longitudinal data and larger population strengthens potential for success.
- Conducting what-if analyses by projecting effects from the 6 states onto 51 individual state ADAPs (50 states and DC) will help evaluate and identify best mix of healthcare delivery programs to achieve the necessary VLS reduction goals.
- In parallel to this proposal, team is collaborating with NASTAD on the annual. Public health survey, that is source of data for this study, to ensure consistent data collection across years, to collect missing data systematically. This could help with long-term monitoring and improvement.

- The findings are expected to inform state ADAPs, advocates, policymakers, and Ending the HIV Epidemic initiative leaders on best practices to develop new interventions (laws, federal guidance, funding) to optimize viral suppression—translating data into policy and action.

Weaknesses

- This dataset does not have data by social determinants, which could be confounders of variations across states.

2. Investigator(s):

Strengths

- Great lead investigators with long-standing experience collaborations studying state ADAPs. Partners at the National Alliance of State & Territorial AIDS Directors (NASTAD),
- Partners include (NASTAD and state health departments, including the state ADAPs with the highest and the lowest viral suppression rates), access to state ADAP client-level data. NASTAD letter from director notes help with recruitment of ADAP leadership across the nation.
- Strong record of publications that also informed policy changes. Previous works resulted in changes to North Carolina laws, making their ADAP similar to Virginia's, given the improved viral suppression associated with Virginia's ADAP-subsidized insurance plans.

Weaknesses

- None.

3. Innovation:

Strengths

- Aggregation of client-level data with HRSA and NASTAD annually report national data on state ADAPs' programmatic decisions, service utilization, and finances, is new. Combining six state ADAPs' statewide client-level data into a multistate cohort is new. States selected have widely varying features and represent 20% of ADAP clients. Would generate rich data for the first time, increasing potential for success of proposed goals.
- Focuses on optimizing sustained viral suppression for a marginalized group, PWH with low incomes who rely on ADAPs. Help to optimize real-world viral suppression by identifying critical problems and quantifying the benefits and harms.

Weaknesses

- None noted by reviewer.

4. Approach:

Strengths

- Use of Causal Inference Framework to Quantify the Impact of the ADAP Healthcare Program Towards Reducing Disparities Related to Race/Ethnicity.
- Mediation Analyses to Decompose Disparities in Viral Suppression: to separate the overall disparities into the controlled direct effect of race/ethnicity, as an imperfect social construct, and the indirect effect of modifiable healthcare delivery factors (ADAP-subsidized insurance plan versus ADAP-provision of ART only), on the binary outcome of HIV viral suppression.

- Using the survey's state-level aggregate client data to determine the individual states' ADAP client mix. To predict how the different ADAP healthcare delivery programs would affect viral suppression for each individual state ADAP for different subpopulations defined by age, gender, race, ethnicity, income, and baseline viral suppression. For each individual state ADAP, study will quantify the number of ADAP clients who need to shift from ADAP-provision of ART only to ADAP-subsidized insurance plans to achieve a 5% improvement in viral suppression.
- Will analyze in-depth semi-structured interviews with state AIDS directors and ADAP directors to identify ADAP programs and policies that help ADAP clients achieve sustained viral suppression and reduce disparities in outcome by race/ethnicity. Analyses will include descriptive statistics, conventional qualitative content analysis and situational mapping, in which themes are derived from data.

Weaknesses

- Study notes will use simulation to project effects from 6 states onto all 50 states to identify relevant mix of delivery programs. It is not clear what will be simulated, and details of it is not present. The method noted seems to be a regression model.
- This dataset does not have data by social determinants, potential confounders for variations across states. Separating out its effects from differences in policies and programs will be done by using other sources where available. Viral suppression data by SDOH subgroups from randomized clinical trials or large cohort studies, or RWAHP will be used to identify magnitude and direction of influence. But how will social determinants be associated with persons receiving the two different ADAP programs is not clear and thus the ability to fully control for the confounding factors.

5. Environment:

Strengths

- Strong, no concerns.

Weaknesses

- None noted by reviewer.

Study Timeline:

Not Applicable (Not Clinical Trial)

Protections for Human Subjects:

Acceptable Risks and/or Adequate Protections

Inclusion Plans:

- Sex/Gender: Distribution justified scientifically.
- Race/Ethnicity: Distribution justified scientifically.
- For NIH-Defined Phase III trials, Plans for valid design and analysis: Not Applicable
- Inclusion/Exclusion Based on Age: Distribution justified scientifically.

Vertebrate Animals:

Not Applicable (No Vertebrate Animals)

Biohazards:
Not Applicable (No Biohazards)

Resubmission:

- The resubmission has been mostly responsive to previous reviews.

Applications from Foreign Organizations:
Not Applicable (No Foreign Organizations)

Select Agents:
Not Applicable (No Select Agents)

Resource Sharing Plans:
Acceptable

Authentication of Key Biological and/or Chemical Resources:
Not Applicable (No Relevant Resources)

Budget and Period of Support:
Recommend as Requested

CRITIQUE 3

Significance: 4
Investigator(s): 3
Innovation: 3
Approach: 4
Environment: 2

Overall Impact: This is a proposal to conduct analyses of the data from six states participating in ADAP to examine the mediators of lower VS among Black participants (Aim 1), to conduct simulations studies to determine what is needed to improve VS in all 50 states + DC (Aim 2), and to conduct qualitative interviews with state ADAP leaders to explore potential improvements. The team is large though the work will mostly be conducted by the PI and one co-I. I have some concern on the framing of Aim 2. The need for extended data collection (through 2024) is not justified, and the application would have higher impact if the Aims 1 and 2 could be completed earlier to impact policy earlier.

1. Significance:

Strengths

- The goal of improving VS among low-income persons is highly significant, especially given the disparity across states and across race/ethnicity.

Weaknesses

- The statement of Aim 2 is odd – if states are behind in VS the goal should be an absolute change in VS, rather than a relative change (5%). Note 5% of 80% is 4, while 5% of 50% is 2.5. So their hypothesis is really that the rate of VS is lower in some states.

2. Investigator(s):

Strengths

- The investigative team is strong. It is a real strength to include persons involved with the ADAP programs as well as a lawyer immersed in policy.

Weaknesses

- There are several investigators with very small % efforts. It would be helpful to streamline this where possible, rather than spreading the team out so thinly.

3. Innovation:

Strengths

- Causal inference with this sort of data is innovative.

Weaknesses

- None noted by reviewer.

4. Approach:

Strengths

- The approach is clearly described.

Weaknesses

- No justification for needing to collect data through 2024, as statistical power does not seem to be an issue.

5. Environment:

Strengths

- The environment is excellent.

Weaknesses

- None noted by reviewer.

Study Timeline:

Strengths

- Steps are clearly defined.

Weaknesses

- It is not clear that data to 2024 are needed – this extends the timeline and delays results.

Protections for Human Subjects:

Acceptable Risks and/or Adequate Protections.

Data and Safety Monitoring Plan (Applicable for Clinical Trials Only):

Acceptable

Inclusion Plans:

- Sex/Gender: Distribution justified scientifically.
- Race/Ethnicity: Distribution justified scientifically.
- For NIH-Defined Phase III trials, Plans for valid design and analysis: Not Applicable.
- Inclusion/Exclusion Based on Age: Distribution justified scientifically.

Vertebrate Animals:

Not Applicable (No Vertebrate Animals)

Biohazards:

Not Applicable (No Biohazards)

Resubmission:

- Responsive to prior reviews.

Applications from Foreign Organizations:

Not Applicable (No Foreign Organizations)

Select Agents:

Not Applicable (No Select Agents)

Resource Sharing Plans:

Acceptable

Authentication of Key Biological and/or Chemical Resources:

Not Applicable (No Relevant Resources)

Budget and Period of Support:

Budget Modifications Recommended (in amount/time)

Recommended budget modifications or possible overlap identified:

- It seems like a large budget and long timeline to accomplish the aims.

THE FOLLOWING SECTIONS WERE PREPARED BY THE SCIENTIFIC REVIEW OFFICER TO SUMMARIZE THE OUTCOME OF DISCUSSIONS OF THE REVIEW COMMITTEE, OR REVIEWERS' WRITTEN CRITIQUES, ON THE FOLLOWING ISSUES:

PROTECTION OF HUMAN SUBJECTS: ACCEPTABLE

INCLUSION OF WOMEN PLAN: ACCEPTABLE

INCLUSION OF MINORITIES PLAN: ACCEPTABLE

INCLUSION ACROSS THE LIFESPAN: ACCEPTABLE

COMMITTEE BUDGET RECOMMENDATIONS: The budget was recommended as requested.

Footnotes for 1 R01 AI170093-01A1; PI Name: McManus, Kathleen Ann

NIH has modified its policy regarding the receipt of resubmissions (amended applications).See Guide Notice NOT-OD-18-197 at https://grants.nih.gov/grants/guide/notice-files/NOT-OD-18-197.html. The impact/priority score is calculated after discussion of an application by averaging the overall scores (1-9) given by all voting reviewers on the committee and multiplying by 10. The criterion scores are submitted prior to the meeting by the individual reviewers assigned to an application, and are not discussed specifically at the review meeting or calculated into the overall impact score. Some applications also receive a percentile ranking. For details on the review process, see http://grants.nih.gov/grants/peer_review_process.htm#scoring.

MEETING ROSTER

Population and Public Health Approaches to HIV/AIDS Study Section
Healthcare Delivery and Methodologies Integrated Review Group
CENTER FOR SCIENTIFIC REVIEW
PPAH
07/14/2022 - 07/15/2022

Notice of NIH Policy to All Applicants: Meeting rosters are provided for information purposes only. Applicant investigators and institutional officials must not communicate directly with study section members about an application before or after the review. Failure to observe this policy will create a serious breach of integrity in the peer review process, and may lead to actions outlined in NOT-OD-22-044 at https://grants.nih.gov/grants/guide/notice-files/NOT-OD-22-044.html, including removal of the application from immediate review.

CHAIRPERSON(S)

YOUNG, APRIL MARIE, MPH, PHD
ASSOCIATE PROFESSOR
DEPARTMENT OF EPIDEMIOLOGY
COLLEGE OF PUBLIC HEALTH
UNIVERSITY OF KENTUCKY
LEXINGTON, KY 40536

MEMBERS

ABUOGI, LISA LYNN, MD
ASSOCIATE PROFESSOR
DEPARTMENT OF PEDIATRICS
SCHOOL OF MEDICINE
UNIVERSITY OF COLORADO, DENVER
AURORA, CO 80045

AMIRKHANIAN, YURI A, PHD
PROFESSOR
DEPARTMENT OF PSYCHIATRY AND BEHAVIORAL MEDICINE
CENTER FOR AIDS INTERVENTION RESEARCH
MEDICAL COLLEGE OF WISCONSIN
MILWAUKEE, WI 53202

ARONSON, IAN DAVID, PHD *
ASSOCIATE RESEARCH SCIENTIST
CENTER FOR DRUG USE AND HIV HCV RESEARCH
SCHOOL OF GLOBAL PUBLIC HEALTH
NEW YORK UNIVERSITY
NEW YORK, NY 10003

BARNIGHAUSEN, TILL, MD
PROFESSOR AND DIRECTOR
HEIDELBERG INSTITUTE OF GLOBAL HEALTH
UNIVERSITY OF HEIDELBERG
HEIDLEBURG, GERMANY 69120
GERMANY

BLANK, MICHAEL B, PHD *
PROFESSOR
DEPARTMENT OF PSYCHIATRY
PERELMAN SCHOOL OF MEDICINE
UNIVERSITY OF PENNSYLVANIA
PHILADELPHIA, PA 19104

BOEKELOO, BRADLEY O, PHD *
PROFESSOR
DEPARTMENT OF BEHAVIORAL AND COMMUNITY HEALTH
SCHOOL OF PUBLIC HEALTH
UNIVERSITY OF MARYLAND
COLLEGE PARK, MD 20742

BUDHWANI, HENNA, PHD *
ASSOCIATE PROFESSOR
DEPARTMENT OF HEALTH CARE ORGANIZATION
AND POLICY
SCHOOL OF PUBLIC HEALTH
THE UNIVERSITY OF ALABAMA AT BIRMINGHAM
BIRMINGHAM, AL 35924

DARBES, LYNAE A, PHD
ASSOCIATE PROFESSOR
DEPARTMENT OF HEALTH BEHAVIOR
AND BIOLOGICAL SCIENCES
SCHOOL OF NURSING
UNIVERSITY OF MICHIGAN
ANN ARBOR, MI 48109

DEGRUTTOLA, VICTOR GERARD, DSC *
PROFESSOR
DEPARTMENT OF BIOSTATISTICS
SCHOOL OF PUBLIC HEALTH
HARVARD UNIVERSITY
BOSTON, MA 02115

EDLAND, STEVEN DYAL, PHD *
PROFESSOR
DEPARTMENT OF FAMILY MEDICINE AND
PUBLIC HEALTH
UNIVERSITY OF CALIFORNIA, SAN DIEGO
LA JOLLA, CA 92093

FOX, MATTHEW ALEXANDER PEASE, DSC, MPH *
PROFESSOR
DEPARTMENT OF EPIDEMIOLOGY
SCHOOL OF PUBLIC HEALTH
BOSTON UNIVERSITY
BOSTON, MA 02118

FUJIMOTO, KAYO, PHD
DISTINGUISHED PROFESSOR
DEPARTMENT OF HEALTH PROMOTION
AND BEHAVIORAL SCIENCES
SCHOOL OF PUBLIC HEALTH
UNIVERSITY OF TEXAS HEALTH SCIENCE CENTER
HOUSTON, TX 77030

GOEDEL, WILLIAM C, PHD *
ASSISTANT PROFESSOR
DEPARTMENT OF EPIDEMIOLOGY
SCHOOL OF PUBLIC HEALTH
BROWN UNIVERSITY
PROVIDENCE, RI 02912

GOPALAPPA, CHAITRA, PHD *
ASSOCIATE PROFESSOR
DEPARTMENT OF MECHANICAL
AND INDUSTRIAL ENGINEERING
COLLEGE OF ENGINEERING
UNIVERSITY OF MASSACHUSETTS, AMHERST
AMHERST, MA 01003

HAHN, JUDITH ALISSA, PHD *
PROFESSOR
DEPARTMENT OF MEDICINE
SAN FRANCISCO GENERAL HOSPITAL
UNIVERSITY OF CALIFORNIA, SAN FRANCISCO
SAN FRANCISCO, CA 94143

HECKMAN, TIMOTHY GLENN, PHD *
PROFESSOR AND ASSOCIATE DEAN
DEPARTMENT OF HEALTH PROMOTION AND BEHAVIOR
COLLEGE OF PUBLIC HEALTH
UNIVERSITY OF GEORGIA
ATHENS, GA 30602

HERBECK, JOSHUA T, PHD *
ASSISTANT PROFESSOR
DEPARTMENT OF GLOBAL HEALTH
UNIVERSITY OF WASHINGTON
SEATTLE, WA 98195

JENNESS, SAMUEL, MPH, PHD *
ASSOCIATE PROFESSOR
DEPARTMENT OF EPIDEMIOLOGY
ROLLINS SCHOOL OF PUBLIC HEALTH
EMORY UNIVERSITY
ATLANTA, GA 30030

KERSHAW, TRACE S, PHD *
PROFESSOR
CENTER FOR INTERDISCIPLINARY RESEARCH ON AIDS
DEPARTMENT OF EPIDEMIOLOGY
SCHOOL OF PUBLIC HEALTH
YALE UNIVERSITY
NEW HAVEN, CT 06510

LAU, BRYAN, PHD *
PROFESSOR
DEPARTMENT OF EPIDEMIOLOGY
BLOOMBERG SCHOOL OF PUBLIC HEALTH
JOHNS HOPKINS UNIVERSITY SCHOOL OF MEDICINE
BALTIMORE, MD 21205

LEITNER, THOMAS K, PHD *
STAFF SCIENTIST
THEORETICAL BIOLOGY AND BIOPHYSICS GROUP
LOS ALAMOS NATIONAL LABORATORY
LOS ALAMOS, NM 87545

MCMAHON, JAMES M, PHD
ASSOCIATE PROFESSOR AND ENDOWED CHAIR
SCHOOL OF NURSING
UNIVERSITY OF ROCHESTER MEDICAL CENTER
ROCHESTER, NY 14642

MEEK, ERIN, DRPH, MPH *
SENIOR RESEARCH SCIENTIST
AIDS OFFICE
SAN FRANCISCO DEPARTMENT OF PUBLIC HEALTH
SAN FRANCISCO, CA 94102

NASH, DENIS, MPH, PHD *
PROFESSOR
DEPARTMENT OF EPIDEMIOLOGY AND BIOSTATISTICS
SCHOOL OF PUBLIC HEALTH
CITY UNIVERSITY OF NEW YORK
NEW YORK, NY 10035

NIJHAWAN, ANK ELISABETH, MD, MPH *
ASSOCIATE PROFESSOR
INTERNAL MEDICINE, DIVISION OF INFECTIOUS DISEASES
UT SOUTHWESTERN MEDICAL CENTER
DALLAS, TX 75390

OUTLAW, ANGULIQUE Y, PHD *
ASSOCIATE PROFESSOR
DEPARTMENT OF FAMILY MEDICINE AND
PUBLIC HEALTH SCIENCES
SCHOOL OF MEDICINE
WAYNE STATE UNIVERSITY
DETROIT, MI 48202

PHO, MAI TUYET, MD, MPH
ASSOCIATE PROFESSOR
DEPARTMENT OF MEDICINE
SECTION OF INFECTIOUS DISEASES AND GLOBAL HEALTH
UNIVERSITY OF CHICAGO MEDICAL CENTER
CHICAGO, IL 60637

RAMIREZ (KITCHEN), CHRISTINA MICHELLE, PHD *
ASSOCIATE PROFESSOR
DEPARTMENT OF BIOSTATISTICS
SCHOOL OF PUBLIC HEALTH
UNIVERSITY OF CALIFORNIA, LOS ANGELES
LOS ANGELES, CA 90095

SALEEM, HANEEFA TASLEEM, MPH, PHD *
ASSISTANT PROFESSOR
BLOOMBERG SCHOOL OF PUBLIC HEATH
JOHNS HOPKINS UNIVERSITY
BALTIMORE, MD 21205

SALEMI, MARCO, PHD
PROFESSOR
DEPARTMENT OF PATHOLOGY, IMMUNOLOGY,
AND LABORATORY MEDICINE
COLLEGE OF MEDICINE
UNIVERSITY OF FLORIDA
GAINESVILLE, FL 32610

SEAL, DAVID W, PHD *
PROFESSOR
DEPARTMENT OF GLOBAL COMMUNITY HEALTH
AND BEHAVIORAL SCIENCES
SCHOOL OF PUBLIC HEALTH AND TROPICAL MEDICINE
TULANE UNIVERSITY
NEW ORLEANS, LA 70112

VARDAVAS, RAFFAELE, PHD *
MATHEMATICIAN
FACULTY PARDEE RAND GRADUATE SCHOOL
RAND CORPORATION
SANTA MONICA, CA 90407

WITTE, SUSAN S, PHD
PROFESSOR
SCHOOL OF SOCIAL WORK
COLUMBIA UNIVERSITY
NEW YORK, NY 10027

MAIL REVIEWER(S)

GOLIN, CAROL E, MD
PROFESSOR
DEPARTMENT OF HEALTH BEHAVIOR AND
HEALTH EDUCATION
UNIVERSITY OF NORTH CAROLINA
CHAPEL HILL, NC 27599

SCIENTIFIC REVIEW OFFICER

GUERRIER, JOSE H, PHD
SCIENTIFIC REVIEW OFFICER
CENTER FOR SCIENTIFIC REVIEW
NATIONAL INSTITUTES OF HEALTH
BETHESDA, MD 20892

* Temporary Member. For grant applications, temporary members may participate in the entire meeting or may review only selected applications as needed.

Consultants are required to absent themselves from the room during the review of any application if their presence would constitute or appear to constitute a conflict of interest.
